# Quantification of a Sulfated Marine-Inspired Antifouling Compound in Several Aqueous Matrices: Biodegradation Studies and Leaching Assays from Polydimethylsiloxane Coatings

**DOI:** 10.3390/md20090548

**Published:** 2022-08-25

**Authors:** Cátia Vilas-Boas, Virgínia Gonçalves, Paolo De Marco, Emília Sousa, Madalena Pinto, Elisabete R. Silva, Maria Elizabeth Tiritan, Marta Correia-da-Silva

**Affiliations:** 1 Laboratory of Organic and Pharmaceutical Chemistry, Department of Chemical Sciences, Faculty of Pharmacy, University of Porto, Rua Jorge Viterbo Ferreira, 228, 4050-313 Porto, Portugal; 2 CIIMAR/CIMAR—Interdisciplinary Center for Marine and Environmental Research, University of Porto, Avenida General Norton de Matos, 4450-208 Matosinhos, Portugal; 3 UNIPRO—Oral Pathology and Rehabilitation Research Unit, University Institute of Health Sciences (IUCS), CESPU, 4585-116 Gandra, Portugal; 4 TOXRUN—Toxicology Research Unit, University Institute of Health Sciences, CESPU, CRL, 4585-116 Gandra, Portugal; 5 BioISI-Biosystems & Integrative Sciences Institute, Faculdade de Ciências, Universidade de Lisboa, 1749-016 Lisboa, Portugal; 6 CERENA-Centro de Recursos Naturais e Ambiente, Instituto Superior Técnico, Universidade de Lisboa, 1049-001 Lisboa, Portugal

**Keywords:** biodegradation, leaching, HILIC, sulfated, polydimethylsiloxane

## Abstract

The development of marine-inspired compounds as non-toxic antifouling (AF) agents has been pursued in the last years. Sulfur is the third most common element in seawater. Sulfur is present in oxygenated seawater as sulfate anion (SO_4_^2−^), which is the most stable combination of sulfur in seawater, and several promising AF secondary metabolites with sulfate groups have been described. However, sulfated compounds proved to be an analytical challenge to quantify by HPLC. Taking these facts into consideration, this work presents the development and validation of a method for the quantification of gallic acid persulfate (GAP) in seawater and ultrapure water matrix, based on hydrophilic interaction liquid chromatography (HILIC). This method was used to evaluate GAP stability following several abiotic and biotic degradation assays, and to quantify its release in seawater from room-temperature-vulcanizing polydimethylsiloxane commercial coating. GAP was very stable in several water matrices, even at different pH values and in the presence/absence of marine microorganisms and presented a leaching value lower than 0.5%. This work discloses HILIC as an analytical method to overcome the difficulties in quantifying sulfated compounds in water matrices and highlights the potential of GAP as a promising long-lasting coating.

## 1. Introduction

Marine organisms, namely microorganisms, micro- and macroalgae, and invertebrates, are extremely rich in sulfated compounds [1,2,3]. Sulfate ions (SO_4_^2−^) are the most stable combination of sulfur in seawater. Over the years, it has been found that several sulfated metabolites are powerful antifouling (AF) agents [2,3]. Therefore, the synthesis of marine-inspired sulfated molecules in the search for environmentally friendly AF compounds has been a fruitful approach [4,5]. One of the most promising marine-inspired sulfated compounds is gallic acid persulfate (GAP), active against the larvae settlement of *Mytilus galloprovincialis*, without exhibiting toxicity against this major macrofouler specie and against other non-target organisms [4]. This probably occurs due to its high polarity and water solubility, which may prevent GAP from being absorbed/accumulated in the adipose tissues of marine organisms (low Log_Koc_) [4]. Despite its high water solubility, GAP was successfully immobilized into several commercial AF coatings, as is the case of polydimethylsiloxane (PDMS)-based coatings [6]. PDMS-based coatings have received increasing interest due to their low surface energy and relatively higher elasticity, reducing the adhesion of organisms and promoting their removal. However, their effectiveness is more pronounced under dynamic conditions, making the combination of these coatings with AF agents a more ideal strategy [7].

However, due to the highly charged properties of this sulfated compound, analysis by high-performance liquid chromatography (HPLC) has always been a challenge since typically, C18 columns do not retain this compound, hampering its quantification [8]. As such, ion-pairing reversed-phase (IP-RP) chromatography has provided for many years a suitable option for the analysis of these complex charged molecules [9]. The downside is that ion-pairing agents change the surface chemistry of the chromatographic column, considerably reducing its lifetime. Additionally, based on our previous experience, the HPLC analysis of sulfated compounds dissolved in seawater matrices has proven to be a difficult task even with the use of a suitable ion-pairing agent as is the case of tetrabutylammonium bromide (TBAB) [6]. Furthermore, ion-pair reagents interfere with the sensitivity of mass spectrometry (MS) detection, making the identification of new products unfeasible [10]. Hydrophilic interaction liquid chromatography (HILIC) has been established as a more suitable and sensitive method for separating (HPLC mode) and detecting (MS mode) small organic acids, basic drugs, and many other neutral, charged, and amphiphilic molecules such as drugs, toxins, plant extracts, and among others, which are important to food and pharmaceutical industries, that are too polar to be retained in reversed-phase liquid chromatographic mode (RP-LC) [8]. Similar to normal-phase liquid chromatography (NP-LC), HILIC columns contain traditional polar stationary phases such as silica, amino, cyano, and cross-linked diol silica. Diol phases typically contain 2,3-dihydroxypropyl ligands, which show both hydrogen donor and hydrogen acceptor activities via the –OH group creating hydrogen bond interactions, in addition to the hydrophilic partitioning, for polar analytes [11,12]. Furthermore, the mobile phase used in these columns is similar to those employed in the RP-LC mode, with a typical mobile phase for HILIC chromatography including water-miscible polar organic solvents such as acetonitrile with a small amount of water [13]. The polar analyte undergoes a partition mechanism between the two liquid phases: the aqueous layer immobilized on the surface of the column and the organic mobile phase. Retention in HILIC is primarily governed by this partitioning mechanism, but other mechanisms such as adsorption and electrostatic interaction may also play a role in the retention [14]. Few reports describe applications of HILIC to sulfated compounds in seawater matrices [15,16].

An analytical methodology using an HILIC column was developed, with the main goal of quantifying this very polar trisulfated compound in several water samples resulting from environmental fate studies. The robustness of the HILIC method allowed us to analyze this very polar trisulfated compound in several water samples, including seawater, ultrapure water, and different buffer samples used in biodegradation studies and leaching waters obtained after submerging GAP-based room-temperature-vulcanizing polydimethylsiloxane (PDMS-RTV) coatings.

## 2. Results

### 2.1. Development and Validation of the HILIC Method

The buffer concentration of the mobile phase was first studied to improve the GAP retention and chromatographic performance. Initially, a concentration of 10 mM of ammonium acetate was used, but no retention was observed. Then, an increase to 20 mM was carried out with several acetonitrile: water (*v*/*v*) proportions (Figure S1). A suitable retention and peak shape of GAP was accomplished with an optimized mobile phase proportion (78:22 *v/v*) of acetonitrile: 20 mM of ammonium acetate in water acidified with acetic acid (pH 5) to avoid hydrolysis of GAP. The temperature was also evaluated and set at 28 °C since lower values (22 and 25 °C) resulted in a long retention time (Figure S2). A retention time of 5.5 and 6.5 ± 0.4 min for GAP dissolved in UPW (Figure S3) and NSW (Figure S4), respectively, was obtained. HILIC aqueous solvents/buffers have a high elution strength, causing substantial peak distortion and a decrease in retention. To avoid this, standard solutions of GAP containing UPW and NSW as matrices were pre-treated with acetonitrile before analysis to achieve successful separation with more effective retention without peak distortion, peak broadening, and earlier elution [17]. A sample dilution of 1:1 with acetonitrile before injection was selected. Due to its low fluorescence intensity (data not shown), GAP was detected with a UV detector (λ_max_ = 236 nm).

The newly developed method using HILIC was compared with our previously developed method, in which a mobile phase containing TBAB (25 mM) was used as ion-pairing, and for NSW matrices, a sample pre-treatment with 60 mM of TBAB in a proportion of 1:3 was applied [6]. The HILIC method accomplished a similar retention time for this sulfated compound dissolved in both water matrices without the need for a different sample treatment for NSW samples. The newly developed method using an HILIC column allowed effective retention of GAP without the use of damaging column buffer solutions as TBAB or pre-treatments for the different water matrices.

The new analytical method for GAP dissolved in UPW and NSW was validated for parameters such as selectivity, linearity, range, accuracy, and precision, according to the ICH Guidance for Industry Q2 (R1) [18]. The developed method was demonstrated to be linear, within the range of 6% to 120% in both matrices.

The LOD and LOQ demonstrated that the sensitivity of the method is higher in the NSW matrix than in the UPW matrix (Table 1). This unusual difference can be explained due to a baseline drift that occurs near the chromatographic signal of the dissolved GAP in UPW (Figure S3), which interferes with signal detection and quantification at very low concentrations.

The accuracy and precision were obtained from the analysis of the quality control solutions (40, 100, 500 µM), with acceptable accuracies (between 80 and 120%) and relative standard deviation (RSD) values for the intra-day and inter-day precision lower than 5% (Table 2).

Overall, the newly developed method was found to be precise, accurate, highly sensitive, and linear in the established range, allowing a suitable quantification of GAP in UPW and NSW matrices without the need to use column-damaging ion-pairing agents. This method could translate to LC-MS analysis to overcome the limitations of polar compounds, such as natural sulfated compounds, polar pharmaceuticals, and their metabolites, in an NSW matrix [19].

### 2.2. Effect of Water pH on GAP Stability

Natural water varies in the number of dissolved minerals, organic matter content, and pH, depending on its source, location, and season. As such, factors such as temperature, solubility, concentration, humidity, and pH can affect the stability of some AF agents. In previous studies, GAP was shown to be stable in sterilized NSW (200 µM) under different temperatures (4, 18, and 25 °C in the absence/presence of light) [6]. Now, using the newly developed HILIC method, the stability of GAP in UPW (500 µM) was assessed under different pH values (5, 7, 9) in the absence of light at 50 °C for a period of 7 days to accelerate hydrolysis [20]. Sterilized NSW was also used for comparative purposes (Table 3).

GAP proved to be stable at different pH values in UPW and sterilized NSW, reinforcing that pH-mediated hydrolysis is not its major degradation pathway.

### 2.3. Biodegradation of GAP in Natural Seawater

Marine microorganisms degrade compounds through enzymatic pathways 200 times faster than photolysis and hydrolysis processes [21]. Therefore, biodegradation of GAP in non-sterilized NSW in the presence or absence of light for a period of 2 months was also studied to provide a more realistic behavior of GAP in natural marine conditions [22]. Microbiological growth was observed in samples that were not subjected to sterilization and in the NSW control. No growth was observed in the matrices that were sterilized and in the sterilized saline solution, confirming the viability of the method and the presence of biotic conditions during the biodegradation assay of GAP.

After 2 months, the concentration of GAP in NSW remained stable both in the presence and absence of light (Table 4). No biotic degradation of GAP was revealed over the two-month incubation.

This result highlights the potential of this water-soluble and stable compound to provide a long service-life for antifouling coatings.

### 2.4. Leaching Assays

Previous studies confirmed the viability of using 0.5 wt.% GAP directly incorporated in a silicone-based PDMS-RTV coating to prevent the attachment of *M. galloprovincialis* larvae, obtaining 0% larvae adhesion after 40 h of exposure [8]. In this work, the GAP release in 45 days of leaching waters, obtained from a PDMS-RTV coating matrix, was evaluated by applying the developed HILIC method. Table 5 shows the amount of GAP detected in those waters. 

The incorporation approach for GAP immobilization in the commercial silicone-based coating succeeded as it allowed GAP leaching values lower than 0.35%. This result demonstrates the good compatibility of GAP with this silicone-based matrix, being in line with a non-release strategy, allowing a higher service life of the coating to be achieved in the future.

## 3. Materials and Methods

### 3.1. Materials

GAP (purity > 95%) was synthesized according to Vilas-Boas et al. (2020) [6]. GAP stock solutions (10 mM) were prepared in ultrapure water (UPW) and stored at −20 °C. UPW was supplied by a Milli-Q water system (Millipore). NSW was collected from Memory Beach (N 41°13′51.5″, W 8°43′15.5″) on the day immediately before starting the degradation studies. Artificial seawater (ASW) was obtained from 33 g/L of sera salt (sera marine salt, Heinsberg, Germany) diluted in distilled water. Acetonitrile HPLC gradient grade was acquired from Honeywell Research Chemicals (Seelze, Germany). Acetic acid, ammonium acetate, sodium acetate, and sodium hydroxide were purchased from ReagentPlus (purity ≥ 99.0%; Sigma-Aldrich (St. Louis, MO, USA), EUA). A Shimadzu UFLC Prominence System equipped with a CBM-20A system controller, two LC-20AD pumps, an SIL-20AC autosampler, a CTO-20AC column oven, and an SPD-20A UV-VIS detector was used. LC Solution software (V. 1.24 SP1, Shimadzu, Kyoto, Japan) was used for data acquisition and analysis. For the chromatographic separation, an HILIC High Purity Silica Gel Inertsil column (3 µm, 150  ×  4.6 mm) (GL Sciences, Tokyo, Japan) was used.

### 3.2. Chromatographic Conditions

The optimized mobile phase consisted of acetonitrile: 20 mM of ammonium acetate in water (78:22, *v/v*). Chromatographic conditions were set at a constant flow rate of 0.8 mL/min in isocratic mode, the injection volume was 10 μL, the column oven temperature was set at 28 °C, and the detection wavelength was set at 236 nm. Before injection, samples were diluted (1:1) in acetonitrile.

### 3.3. Method Validation

The developed HILIC method was validated according to the ICH Guidance for Industry Q2 (R1) through several parameters, namely selectivity, linearity, precision, accuracy, range, limit of detection (LOD), and quantification (LOQ) [18]. To evaluate the linearity and range of the method, six different standard solutions of GAP dissolved in UPW and NSW in the expected working range were prepared: 30, 75, 150, 300, 400, and 600 µM, with three replicates for each concentration. The linearity of several standard samples was analyzed by performing regression analysis using the method of the least squares to obtain a coefficient of determination (R^2^) ≥ 0.98. The accuracy and intra- and inter-day precision of the method were measured through quality control samples prepared at three different concentrations: 40, 100, and 500 µM, with three replicates for each concentration and analyzed on three different days. Accuracy was calculated from the measured concentration/spiked concentration × 100. For precision, the relative standard deviation (RSD) was calculated from 100 × the measured standard deviation (SD)/mean values. LOD and LOQ were calculated based on the signal-to-noise approach by comparing measured signals from standard samples with known low concentrations of each analyte with those of blank samples and establishing the signal-to-noise ratio between 3:1 (LOD) and 10:1 (LOQ).

### 3.4. Degradation Assays

#### 3.4.1. Effect of Water pH on GAP Stability

Experiments were carried out according to the methods described in ASTM (2008) and Huanga et al. (2014) [20,23]. Buffer solutions consisting of 20 mM sodium acetate were prepared and the pH was adjusted to pH 5, 7, or 9 with acetic acid or sodium hydroxide (1 M). Sterilized NSW, pH 7.6, was used for comparative purposes. Each of the four solutions (12 mL) was spiked with GAP stock solution to obtain a final concentration of 500 µM and the tubes were tightly capped and kept in an incubator (KS 4000 ic control, IKA, Königswinter, Germany) with constant agitation (120 rpm) at 50 °C in the dark. At selected time intervals (0, 1, 2, and 7 days), 0.5-mL aliquots were collected and injected into the HPLC system after 1:1 dilution in acetonitrile. Each condition was performed in triplicate.

#### 3.4.2. Biodegradation of GAP in Natural Seawater (NSW)

Glass flasks containing 20 mL of NSW (pH 7.6) were spiked with GAP stock solution to obtain a final concentration of 500 µM each and the flasks were tightly capped. Biologically inactivated samples (sterile NSW) were generated by autoclaving (120 °C, 30 min) a fraction of the collected NSW. A control experiment was set up similarly but without spiked GAP. Half of the glass samples were covered with aluminum foil to avoid contact with light and set in an incubator (KS 4000 ic control, IKA, Königswinter, Germany) with constant agitation (120 rpm) at 25 °C. At selected time intervals (0, 1, 2, 5, 7, 9, 30, and 60 days), 0.5-mL aliquots were taken and injected into the HPLC system after a 1:1 dilution in acetonitrile. Each condition was performed in triplicate. After the assay, the presence of microorganisms in NSW was determined through the number of colony-forming units (CFU) by plating the samples of several biodegradation conditions and successive dilution with a saline solution (1:10, 1:100) in a simple micro-growth assay using R2A agar medium, a low-nutrient medium recommended in standard methods for heterotrophic plate counts.

### 3.5. GAP Incorporation in a Silicone-Based Commercial Coating

GAP was immobilized in a silicone-based commercial coating, room-temperature-vulcanizing polydimethylsiloxane (RTV11, MOMENTIVE, Waterford, Saratoga, NY, USA), following conventional direct incorporation (DI) and chemical incorporation (CI) strategies according to a previously developed methodology [6].

#### Leaching Analysis

GAP released from coatings was assessed using a leaching stirring test method, followed by collection and an extraction step of the obtained leaching waters, as detailed and described elsewhere [8]. In brief, acrylic-coated plates (3.5 × 6 cm) coated with the developed RTV-PDMS coating systems, with or without incorporation, were immersed in ASW for 45 days. The obtained leaching water samples were then submitted to an OASIS^®^ WAX 6cc cartridge according to the previously developed extraction procedure, which allowed 100% recovery [8], and analyzed using the developed HILIC method.

## 4. Conclusions

Some marine sulfated specialized metabolites have emerged as antifoulants with low or nontoxic effects on the environment, as is the case of zosteric acid [3] and several carboxylated sterol sulfates [2]. For this reason, more simple marine-inspired synthetic sulfated compounds have arisen, showing potential as promising biocide substitutes [4]. The development of suitable analytical methods is of extreme importance to overcome the limitations of the analysis of these polar compounds in marine matrices.

The newly developed and validated HPLC method using an HILIC column provided a suitable alternative to damaging ion-pairing agents, allowing the quantification of a highly polar compound, gallic acid persulfate, a promising marine-inspired antifouling compound, with suitable retention times and sensitive detections. The validated method allowed monitoring of the stability of GAP in abiotic and biotic water matrices and the conclusion that GAP leaching minimization was successfully accomplished when GAP was incorporated in the RTV-PDMS polymeric matrix as a result of its high compatibility [8]. Antifouling paints used to prevent biofouling on underwater surfaces are constantly releasing copper and other biocides into the oceans. As a result, the marine industry is facing rigid environmental regulations on the use of coatings with a biocide-releasing mechanism. AF paints that are meant to end up in the oceans cannot be part of a sustainable future. The overall results presented herein put forward the potential of GAP as a long-lasting antifouling agent.

## Figures and Tables

**Table 1:** Linear regression and sensitivity data of the newly developed method using an HILIC column.

Matrix	Range (µM) ^1^	Linear Regression	R^2^	LOD (µM)	LOQ (µM)
UPW	30–600	y = 2013.2x − 35215	0.9994	15	30
NSW		y = 2607.9x + 7506.6	1	0.75	7.5

LOD: limit of detection; LOQ: limit of quantification; NSW: natural seawater; UPW: ultrapure water. ^1^ Analyses were carried out in triplicate.

**Table 2:** Accuracy and intra- and inter-day variability (precision) of the newly developed method using an HILIC column.

Matrix	Concentration (µM) ^1^	Accuracy (% ± SD)	Intra-Day Variability (RSD ± SD)	Inter-Day Variability (RSD ± SD)
UPW	40	116.50 ± 3.1	3.9 ± 1.1	4.9 ± 0.4
	100	103.06 ± 2.5	2.4 ± 0.7	2.9 ± 0.7
	500	99.93 ± 1.5	4.0 ± 2.1	2.9 ± 0.9
NSW	40	113.25 ± 1.0	3.1 ± 1.7	4.8 ± 2.9
	100	118.21 ± 1.7	3.0 ± 1.5	2.5 ± 1.1
	500	111.64 ± 2.2	3.7 ± 1.2	2.6 ± 1.0

NSW: natural seawater; SD: standard deviation; RSD: relative standard deviation; UPW: ultrapure water. ^1^ Mean values ± standard deviation of three independent experiences.

**Table 3:** Influence of pH on GAP stability in water (500 µM) after 7 days at 50 °C (HILIC).

Stress Conditions ^1^	Initial Concentration (%)	Concentration after 1 Day (%)	Concentration after 2 Days (%)	Concentration after 7 Days (%)
SNSW, pH 7.6	100 ± 1.7	97.4 ± 8.8	95.0 ± 4.0	94.8 ± 15.8
UPW, pH 5	100 ± 5.2	95.5 ± 5.4	99.5 ± 1.6	98.6 ± 3.9
UPW, pH 7	100 ± 3.0	98.3 ± 2.6	99.8 ± 1.5	101.3 ± 3.2
UPW, pH 9	100 ± 8.8	97.6 ± 3.7	101.3 ± 3.2	103.9 ± 4.3

SNSW: sterilized natural seawater; UPW: ultrapure water. ^1^ Mean values ± SD of three independent experiences.

**Table 4:** Biodegradation of GAP in natural seawater after 2 months at 25 °C (HILIC).

**Conditions**	**Initial Concentration (%)** **^1^**	**Concentration after 1 Month (%)** **^1^**	**Concentration after 2 Months (%)** **^1^**
Light and non-sterile	100.0 ± 1.3	99.7 ± 1.8	110.9 ± 3.4
Dark and non-sterile	100.0 ± 0.5	100.0 ± 0.8	103.7 ± 1.5
Light and sterile	100.0 ± 0.5	100.3 ± 2.1	108.9 ± 3.6
Dark and sterile	100.0 ± 0.6	102.1 ± 1.6	115.8 ± 3.4

^1^ Mean values ± SD of three independent experiences.

**Table 5:** Quantification of GAP leached from silicone-based coatings after 45 days immersed in artificial seawater (0.5 L) (HILIC).

Coating Matrix	GAP Content in Coating Formulation (wt.%) ^1^	GAP Amount in Coated Plates (mg) ^1^	Amount of Detected GAP in Leaching Waters (mg) ^1^	Content of Released GAP from the Coating (%) ^1^
PDMS-RTV	0.56 ± 0.02	10.4 ± 0.5	0.037 ± 0.004	0.35 ± 0.027

GAP: gallic acid persulfate; PDMS-RTV: room-temperature-vulcanizing polydimethylsiloxane; PVC: polyvinyl chloride. ^1^ Mean values ± SD of two independent experiences.

## Data Availability

Not applicable.

## Supplementary Materials

The following supporting information can be downloaded at: https://www.mdpi.com/article/10.3390/md20090548/s1. Figure S1: Representative chromatogram of GAP-standard solution (500 µM) in natural seawater (NSW) with several mobile phases proportions; Figure S2: Representative chromatogram of GAP-standard solution (500 µM) in natural seawater (NSW) with several column temperatures; Figure S3: Representative chromatogram of standard solutions of GAP (30–600 µM) in ultra-pure water (UPW); Figure S4: Representative chromatogram of standard solutions of GAP (30–600 µM) in natural seawater (NSW).
