# Supplementary material for: Quantification of a Sulfated Marine-Inspired Antifouling Compound in Several Aqueous Matrices: Biodegradation Studies and Leaching Assays from Polydimethylsiloxane Coatings
Source: Mar Drugs. 2022 Aug 25;20(9):548. doi: 10.3390/md20090548 (PMC9506548; doi:10.3390/md20090548)
Supplement: Supplementary file 1 [file marinedrugs-20-00548-s001.zip › marinedrugs-1862280-supplementary.pdf]

Supplementary Material

Quantification of a sulfated marine inspired antifouling compound in several aqueous matrices: biodegradation studies and leaching assays from polydimethylsiloxane coatings

Cátia Vilas-Boas[1,2], Virgínia Gonçalves[3,4], Paolo De Marco[3,4], Emília Sousa[1,2], Madalena Pinto[1,2], Elisabete R. Silva[5,6], Maria Elizabeth Tiritan[1,2,3,4,*] and Marta Correia-da-Silva[1,2,*]

1 Laboratory of Organic and Pharmaceutical Chemistry, Department of Chemical Sciences, Faculty of Pharmacy, University of Porto, Rua Jorge Viterbo Ferreira, 228, 4050-313 Porto, Portugal
2 CIIMAR/CIMAR— Interdisciplinary Center for Marine and Environmental Research, University of Porto, Avenida General Norton de Matos, 4450-208 Matosinhos, Portugal
3 UNIPRO – Oral Pathology and Rehabilitation Research Unit, University Institute of Health Sciences (IUCS), CESPU, 4585-116 Gandra, Portugal
4 TOXRUN – Toxicology Research Unit, University Institute of Health Sciences, CESPU, CRL, Gandra, 4585-116, Portugal
5 BioISI-Biosystems & Integrative Sciences Institute, Faculdade de Ciências, Universidade de Lisboa, 1749-016 Lisboa, Portugal
6 CERENA-Centro de Recursos Naturais e Ambiente, Instituto Superior Técnico, Universidade de Lisboa, 1049-001 Lisboa, Portugal
* Correspondence: beth@ff.up.pt (M.E.T.); m_correiadasilva@ff.up.pt (M.C.-d.-S.)

Index

Figure S1. Representative chromatogram of GAP-standard solution (500 μM) in natural seawater (NSW) with several mobile phases proportions……………………………………………………….……………..…….................. 3

Figure S2. Representative chromatogram of GAP-standard solution (500 μM) in natural seawater (NSW) with several column temperatures……....…………………………………………………….………….....…….. 3

Figure S3. Representative chromatogram of standard solutions of GAP (30-600 μM) in ultra-pure water (UPW)……………………………………………..…………………...…………………….……………………...... 4

Figure S4. Representative chromatogram of standard solutions of GAP (30-600 μM) in natural seawater (NSW)……………………………………………......………………....…….……………….....………………….. 4

Chromatographic analysis of GAP

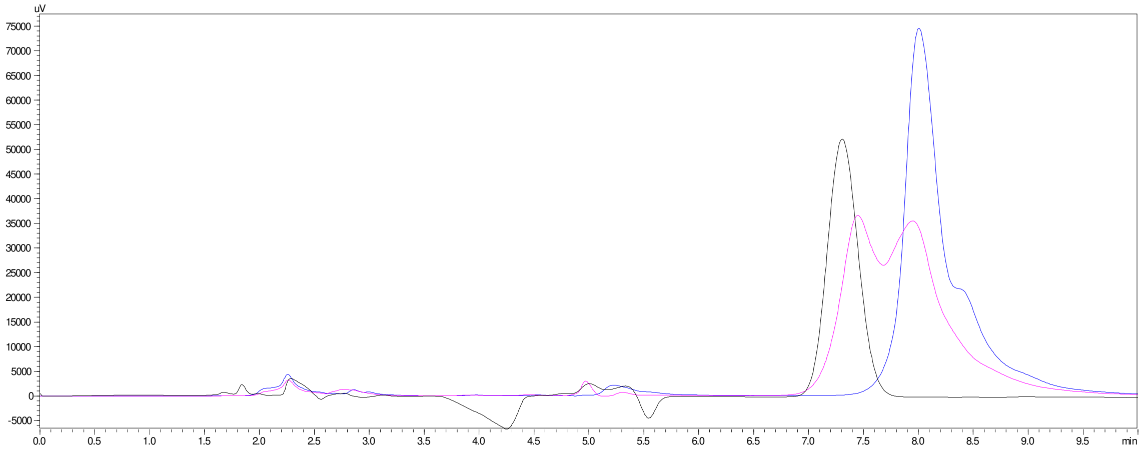

Figure S1. Representative HPLC chromatogram of GAP-standard solution (500 μM) in natural seawater (NSW), diluted 1:1 before injection with acetonitrile and several proportions of acetonitrile: 20 mM aqueous ammonium acetate as mobile phase, namely (78:22 v/v, black line), (50:50 v/v, pink line), and (80:20 v/v, blue line); flow rate at 0.8 mL/min; column: INERTSIL HILIC (3 μm, 150 × 4.6 mm); detection λmax =236 nm.

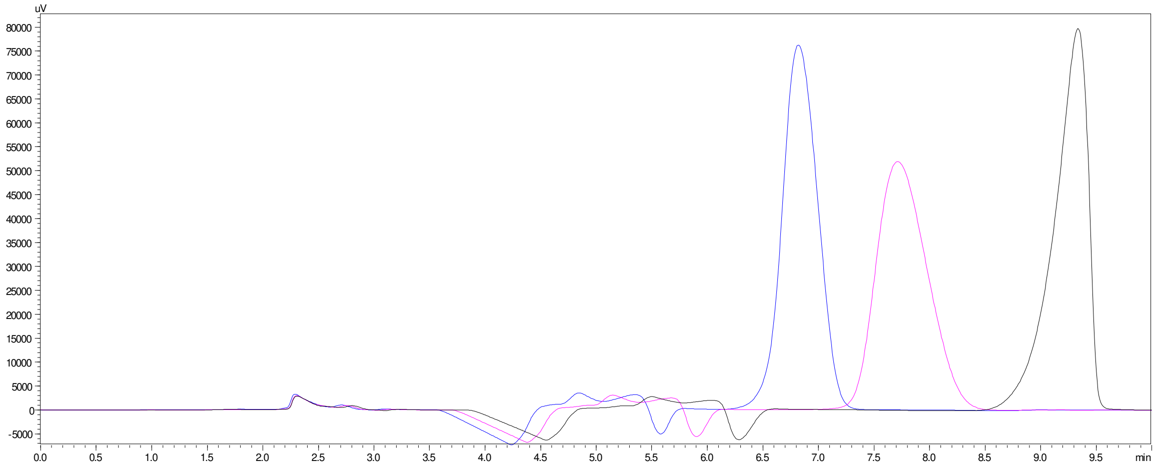

Figure S2. Representative HPLC chromatogram of GAP-standard solution (500 μM) in natural seawater (NSW), diluted 1:1 before injection with acetonitrile and several column temperatures (28 °C, blue line), (25 °C, pink line), (22 °C, black line); Mobile phase: acetonitrile: 20 mM aqueous ammonium acetate (78:22 v/v) with; flow rate at 0.8 mL/min; column: INERTSIL HILIC (3 μm, 150 × 4.6 mm); detection λmax =236 nm.

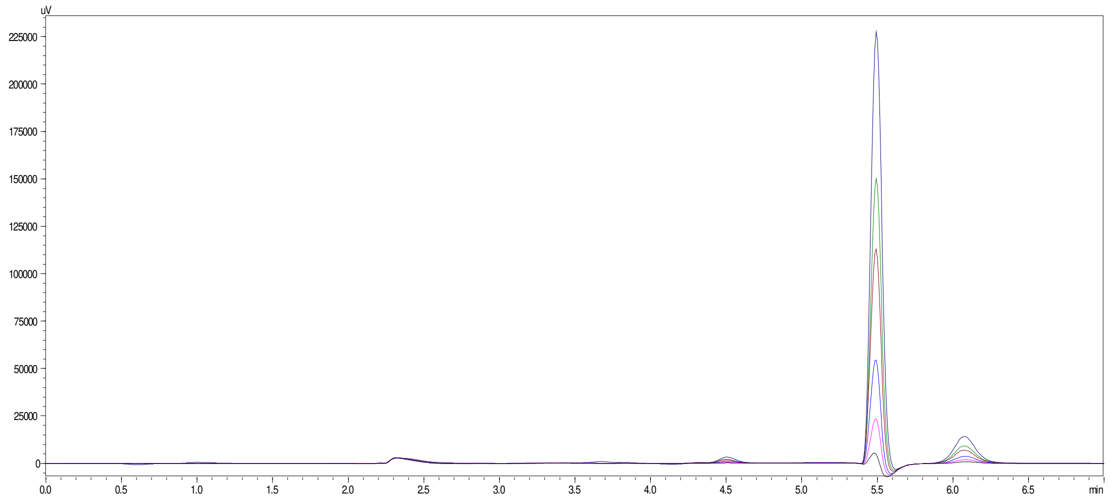

Figure S3. Representative HPLC chromatogram of standard solutions of GAP (30-600 μM) in ultra-pure water (UPW), diluted 1:1 before injection with acetonitrile. Mobile phase: acetonitrile: 20 mM aqueous ammonium acetate (78:22 v/v) with; flow rate at 0.8 mL/min; column: INERTSIL HILIC (3 μm, 150 × 4.6 mm) at 28 ℃; detection λmax =236 nm.

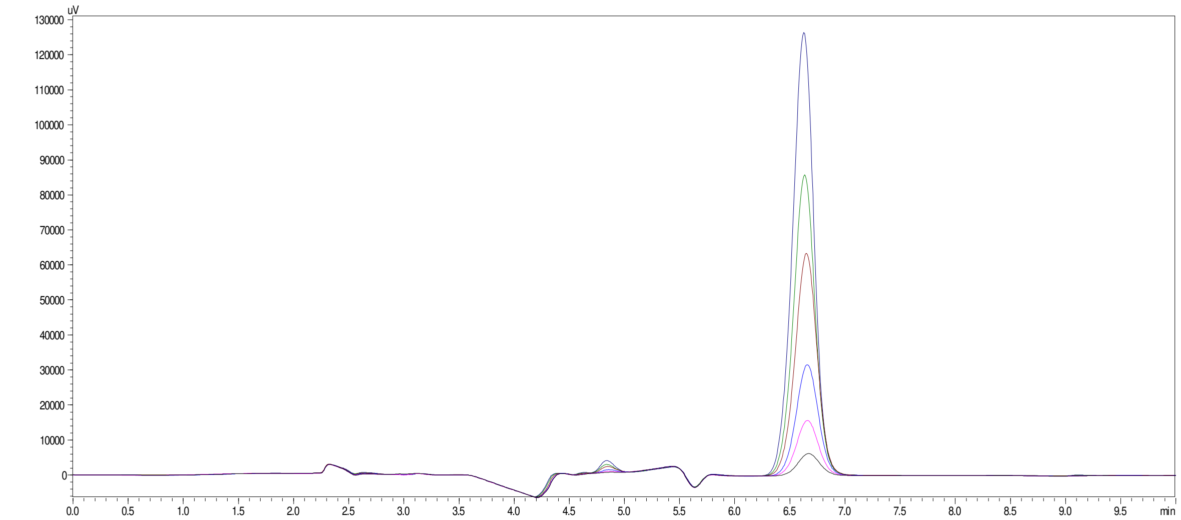

Figure S4. Representative HPLC chromatogram of standard solutions of GAP (30-600 μM) in natural seawater (NSW), diluted 1:1 before injection with acetonitrile. Mobile phase: acetonitrile: 20 mM aqueous ammonium acetate (78:22 v/v) with; flow rate at 0.8 mL/min; column: INERTSIL HILIC (3 μm, 150 × 4.6 mm) at 28 ℃; detection λmax =236 nm.
